# Selection of mosquito life-histories: a hidden weapon against malaria?

**DOI:** 10.1186/1475-2875-11-106

**Published:** 2012-04-03

**Authors:** Heather M Ferguson, Nicolas Maire, Willem Takken, Issa N Lyimo, Olivier Briët, Steve W Lindsay, Thomas A Smith

**Affiliations:** 1 Boyd Orr Centre for Population and Ecosystem Health, University of Glasgow, Glasgow G12 8Q, UK; 2 Department of Epidemiology and Public Health, Swiss Tropical and Public Health Institute, PO Box, CH-4002, Basel, Switzerland; 3 University of Basel, PO Box, CH-4003, Basel, Switzerland; 4 Laboratory of Entomology, Wageningen University, 6700 EH Wageningen, The Netherlands; 5 Biomedical and Environmental Thematic Group, Ifakara Health Institute, Ifakara, PO Box 53, Tanzania; 6 School of Biological and Biomedical Sciences, Durham University, Durham DH1 3LE, UK

**Keywords:** *Anopheles *vectors, Life history evolution, Malaria, Insecticide-treated bed nets, Extrinsic mortality, Natural selection

## Abstract

**Background:**

There has recently been a substantial decline in malaria incidence in much of Africa. While the decline can clearly be linked to increasing coverage of mosquito vector control interventions and effective drug treatment in most settings, the ubiquity of reduction raises the possibility that additional ecological and associated evolutionary changes may be reinforcing the effectiveness of current vector control strategies in previously unanticipated ways.

**Presentation of hypothesis:**

Here it is hypothesized that the increasing coverage of insecticide-treated bed nets and other vector control methods may be driving selection for a shift in mosquito life history that reduces their ability to transmit malaria parasites. Specifically it is hypothesized that by substantially increasing the extrinsic rate of mortality experienced in vector populations, these interventions are creating a fitness incentive for mosquitoes to re-allocate their resources towards greater short-term reproduction at the expense of longer-term survival. As malaria transmission is fundamentally dependent on mosquito survival, a life history shift in this direction would greatly benefit control.

**Testing the hypothesis:**

At present, direct evaluation of this hypothesis within natural vector populations presents several logistical and methodological challenges. In the meantime, many insights can be gained from research previously conducted on wild *Drosophila *populations. Long-term selection experiments on these organisms suggest that increasing extrinsic mortality by a magnitude similar to that anticipated from the up-scaling of vector control measures generated an increase in their intrinsic mortality rate. Although this increase was small, a change of similar magnitude in *Anopheles *vector populations would be predicted to reduce malaria transmission by 80%.

**Implications of hypothesis:**

The hypothesis presented here provides a reminder that evolutionary processes induced by interventions against disease vectors may not always act to neutralize intervention effectiveness. In the search for new intervention strategies, consideration should be given to both the potential disadvantages and advantages of evolutionary processes resulting from their implementation, and attempts made to exploit those with greatest potential to enhance control.

## Background

There has recently been a substantial decline in malaria incidence in Africa [1,2]. Some of the decline can be explained by the massive deployment of insecticide-treated bed nets (ITNs), the introduction of artemisinin combination therapy (ACT), or in some places, by the use of indoor residual spraying (IRS). But these intervention effects alone do not explain all the changes that have been seen. In some places, vector populations have fallen, despite the absence of organized vector control programmes [3], while in others there has been no decline at all [4].

Scaling-up of interventions creates the risk that parasites will develop resistance to drugs [5,6], or that vectors will develop chemical [7,8] or behavioural resistance [9-11]. However, not all selective pressures generated by interventions make malaria control more difficult. For instance, there is some evidence that parasites can be selected for renewed sensitivity to chloroquine [12]; albeit potentially on a short-term basis. Other pressures placed upon mosquito vectors by the rapid scaling-up of ITNs and IRS may have potential to drive selection for new behaviours or phenotypes that could reduce their capacity to transmit.

## Presentation of hypothesis

The major African vectors *Anopheles gambiae *sensu latu and *Anopheles funestus *have adapted over many generations to feed on sleeping humans in mud-brick dwellings, and then rest indoors while digesting their blood meal. This behaviour is under threat from the massive scaling-up of ITNs and IRS [2] which makes host-seeking and resting inside houses far more hazardous for mosquitoes. Concurrently, economic development is improving living conditions in many African countries, and increasing the prevalence of houses with corrugated-iron roofs, brick walls and screened windows that are more difficult for mosquitoes to enter and provide poor resting places for them [13]. These factors combine to increase the fitness costs associated with feeding on humans (anthropophagy), and have potential to generate selection for a shift towards feeding on non-human animals and/or biting and resting outdoors[14-16]. Such shifts may be especially likely if the fitness costs associated with adopting such novel phenotypes are minor [17].

An additional, previously unconsidered and potentially beneficial evolutionary consequence of control measures targeted at the main anthropophilic vectors of malaria in Africa is the selection for a shift in life history that reduces their ability to sustain parasite transmission. Like most organisms, mosquito vectors face trade-offs between investment in reproduction and survival [18], with malaria parasites being intimately dependent on the latter. This is because parasites, such as *Plasmodium falciparum*, require at least 12 days for development inside their vectors before they can infect a new host [19]. However, mosquito longevity in the wild is often low, with < 10% of *An. gambiae *sensu strictu females surviving long enough for parasites to complete their incubation [20]. Consequently any mosquito life-history shift in favour of short-term reproduction at the expense of longer-term survival would greatly reduce mosquito transmission potential. Evolutionary-based approaches to reduce malaria transmission by targeting mosquito survival have been recently proposed in the context of late-life acting insecticides [e.g. [21-23]]. This approach is thematically linked to the hypothesis presented here in its general aim of deploying vector control interventions that do not prompt a detrimental evolutionary response in mosquitoes that would hinder control (e.g. insecticide resistance). However these approaches differ in that the 'evolution-proof' approach requires that the control approach places little or no selection on mosquitoes, whereas the current hypothesis requires the generation of selection but in the direction of reducing intrinsic mosquito survival.

## Testing the hypothesis

Evolutionary theory [24,25] and empirical studies demonstrate that increased exposure to sources of extrinsic mortality can generate selection for increased intrinsic mortality [26,27]. In brief, if external factors make the odds of long life minimal then organisms would be pushed to prioritize early reproduction at the expense of longevity. Laboratory studies have demonstrated that this phenomenon can occur in arthropods exposed to life-shortening pathogens [28]. By similarly increasing extrinsic mortality, the dissemination of insecticidal interventions could thus also place intense selection on malaria vectors for increased intrinsic mortality. Since malaria transmission is highly sensitive to the survival of the adult female mosquito, even a small increase in intrinsic mortality could have profound epidemiological benefits [29]. Indeed, this represents the theoretical rationale for the use of both IRS and ITNs as tools for reducing malaria transmission.

Unfortunately, testing this hypothesis is not easy. A comparison of the fecundity and intrinsic mortality of different wild-caught mosquito populations in relation to the extrinsic mortality rates would be logistically challenging. Recent progress in the establishment of semi-field systems for experimental study of mosquito ecology [e.g. [30]] may increase the feasibility of comparing intrinsic mortality in progeny of wild-caught mosquitoes. However, estimating the relative contribution of intra-specific genetic variation to geographical differences in mosquito mortality, given their ecological and taxonomic complexity, will remain a challenge. As the methodological approaches for study of these phenomena in wild vector populations develops, some a priori hypotheses can be generated from research previously conducted on wild *Drosophila *populations [26]; dipterans often used as model organisms for *Anopheles *genetics and physiology.

**Figure 1:**
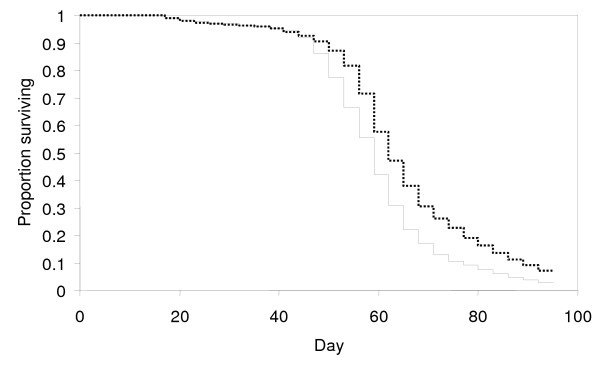
**Kaplan-Meier curves comparing intrinsic mortality of *Drosophila *populations after selection**. Dashed line: low extrinsic mortality selection regime; continuous line: high extrinsic mortality selection regime. Data replotted from [26].

**Figure 2:**
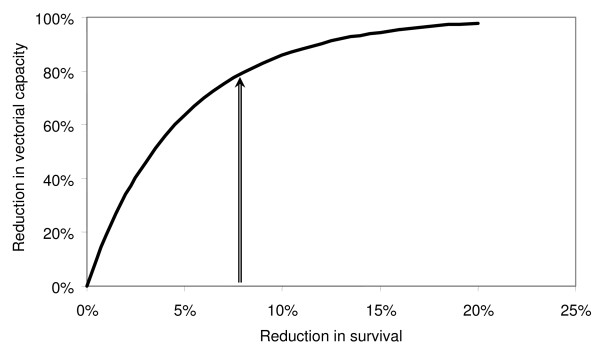
**Predicted relationship between reductions in adult malaria vector survival and their transmission potential**. The reduction in vectorial capacity was calculated using Garrett-Jones' original formula for the vectorial capacity, assuming a daily survival of 90% in the original vector population, and a 12-day duration of malaria sporogony. The vertical arrow corresponds to a 7.7% reduction in survival.

In a series of long-term selection experiments conducted in an insectary, Stearns and colleagues [26] exposed a variety of strains of *Drosophila *to different extrinsic mortality regimes over a period of five years (50-90 generations). Selection under high extrinsic mortality resulted in both an increase in fecundity, and a small increase in (intrinsic) mortality when assayed in the absence of selection (Figure 1)[26]. While for the high intrinsic mortality lines the median survival time (58.4 days) was only 7.7% lower than that of the low intrinsic mortality lines (63.3 days), this difference in relative survival would correspond to an approximately 80% reduction in transmission in a typical endemic setting (Figure 2) given the non-linear relationship between mosquito survival and the vectorial capacity described originally by Garrett-Jones [31], who showed that within the Ross-MacDonald model a reduction in daily survival probability of the vector from *p_1 _*to *p_2 _*results in a reduction in vectorial capacity from *C_1 _*to *C_2 _*as:

$$\frac{C_{2}}{C_{1}}={\left(\frac{p_{2}}{p_{1}}\right)}^{n}\frac{\log_{e}\left(p_{1}\right)}{\log_{e}\left(p_{2}\right)}$$

where *n *is the number of days required for the parasite to complete its extrinsic incubation period [31]. On this basis it appears possible that increased mosquito extrinsic mortality induced by the high coverage of long lasting insecticide treated nets (LLINs) that has now been achieved in many parts of sub-Saharan Africa could select for increased intrinsic mortality with substantial impact on malaria transmission over and above the immediate protection that LLINs provide to communities.

## Implications of hypothesis

The theoretical possibility that such selection could occur does not demonstrate that it is playing a role in recent reductions in malaria transmission in Africa. Amongst other considerations, the magnitude of the hypothesized survival effect depends on all other epidemiologically-relevant aspects of vector ecology remaining the same (e.g. the vector-human ratio, blood feeding rate on humans, duration of sporogony). Any correlated change in these parameters prompted by intervention use could magnify or diminish the transmission effects proposed here. For example, the enhanced investment in short-term reproduction hypothesized here could be manifested as an increase in the number of eggs laid in one clutch. This could intensify competition during larval development, which through its connection with mosquito population growth [32] and adult survival [33] might prompt even greater transmission reduction. Proof of the existence and epidemiological importance of such a phenomenon will require a multi-pronged approach including at least: (1) confirmation of the heritability of intrinsic mortality and reproductive schedule in wild vector populations [34]; (2) evidence of standing genetic variation in these traits; (3) demonstration that the fitness costs imposed by these interventions are of sufficient magnitude to alter the fecundity-longevity trade-off; and (4) development of better tools for tracking the age and fecundity of mosquitoes in natural populations, and their response to increases in vector control coverage. Furthermore, as some empirical studies have shown that extrinsic mortality pressure can select for a net increase in lifetime reproductive success in the absence of pressure [35], caution would be required to ensure that any epidemiological advantages arising from increased vector mortality would not be undermined by an upsurge in the size of their populations.

The possibility of the phenomena described here has important consequences. It provides a reminder that the evolutionary processes induced by interventions against disease vectors may not always act to neutralize intervention effectiveness. Secondly, it argues that large-scale distribution of highly effective interventions could have unpredictable effects. The selective pressures on life history traits depend on local ecology, for example, mosquitoes with high innate fecundity may have the greatest advantage in areas of abundant rainfall where larval habitat is not limiting, whereas in areas with long dry seasons selection may favour mosquito longevity over short-term reproduction [36]. Mosquito evolutionary responses to insecticidal interventions against malaria (e.g. insecticide resistance) have been correlated with a reduction in their ability to transmit other pathogens [37]; suggesting that the selection imposed by these measures could have potentially numerous unanticipated effects on disease risk. The complexity of the parasite-vector-human-environment system will continue to present challenges for prediction. In the search for new control strategies, wide consideration should be given to both the potential epidemiological disadvantages and advantages of evolutionary processes resulting from their implementation.

## Competing interests

The authors declare that they have no competing interests.

## Authors' contributions

All authors contributed to the development of this hypothesis, and provided comments on the manuscript. HMF and TAS drafted the manuscript. All authors read and approved the final manuscript.
